# *Spag16*, an Axonemal Central Apparatus Gene, Encodes a Male Germ Cell Nuclear Speckle Protein that Regulates SPAG16 mRNA Expression

**DOI:** 10.1371/journal.pone.0020625

**Published:** 2011-05-31

**Authors:** David R. Nagarkatti-Gude, Ruth Jaimez, Scott C. Henderson, Maria E. Teves, Zhibing Zhang, Jerome F. Strauss

**Affiliations:** 1 Department of Biochemistry and Molecular Biology, Virginia Commonwealth University, Richmond, Virginia, United States of America; 2 Department of Obstetrics and Gynecology, Virginia Commonwealth University, Richmond, Virginia, United States of America; 3 Center for Research on Reproduction and Women's Health, University of Pennsylvania, Philadelphia, Pennsylvania, United States of America; 4 Department of Anatomy and Neurobiology, Virginia Commonwealth University, Richmond, Virginia, United States of America; Baylor College of Medicine, United States of America

## Abstract

*Spag16* is the murine orthologue of *Chlamydomonas reinhardtii* PF20, a protein known to be essential to the structure and function of the “9+2” axoneme. In *Chlamydomonas*, the *PF20* gene encodes a single protein present in the central pair of the axoneme. Loss of PF20 prevents central pair assembly/integrity and results in flagellar paralysis. Here we demonstrate that the murine *Spag16* gene encodes two proteins: 71 kDa SPAG16L, which is found in all murine cells with motile cilia or flagella, and 35 kDa SPAG16S, representing the C terminus of SPAG16L, which is expressed only in male germ cells, and is predominantly found in specific regions within the nucleus that also contain SC35, a known marker of nuclear speckles enriched in pre-mRNA splicing factors. SPAG16S expression precedes expression of SPAG16L. Mice homozygous for a knockout of SPAG16L alone are infertile, but show no abnormalities in spermatogenesis. Mice chimeric for a mutation deleting the transcripts for both SPAG16L and SPAG16S have a profound defect in spermatogenesis. We show here that transduction of SPAG16S into cultured dispersed mouse male germ cells and BEAS-2B human bronchial epithelial cells increases SPAG16L expression, but has no effect on the expression of several other axoneme components. We also demonstrate that the *Spag16L* promoter shows increased activity in the presence of SPAG16S. The distinct nuclear localization of SPAG16S and its ability to modulate *Spag16L* mRNA expression suggest that SPAG16S plays an important role in the gene expression machinery of male germ cells. This is a unique example of a highly conserved axonemal protein gene that encodes two protein products with different functions.

## Introduction

The “9+2” axoneme, a cytoskeletal structure found in motile cilia and flagella, is composed of nine outer doublet microtubules linked to a central microtubule pair via dynein arms to form a motor complex allowing coordinated force generation [1], [2]. The central pair of microtubules is critical to the integrity and the motility of this structure. One essential element of the central apparatus, PF20, was first identified in *Chlamydomonas rheinhardtii* [3], [4], and has since been shown to exhibit strong conservation amongst a wide variety of organisms and ciliated cell types [5], [6].

We have reported that the murine orthologue of *PF20*, *Spag16*, encodes two distinct proteins: SPAG16L, which is a component of the axoneme central apparatus [7], and SPAG16S, a smaller protein representing the WD repeat region of SPAG16L, identified only in male germ cells [8]. Chimeric mice carrying a mutation that disrupted the *Spag16* gene at a locus shared by transcripts encoding both SPAG16L and SPAG16S displayed a phenotype of haploinsufficiency; the mutant allele was never transmitted to offspring by chimeric males [8]. Furthermore, these mice exhibited significant germ cell loss at the round spermatid stage. In contrast, transgenic mice homozygous for a deleterious mutation in the SPAG16L-specific region of the gene were infertile, with normal spermatogenesis but resulting in sperm showing marked motility defects despite an axonemal structure devoid of significant ultrastructural defects [9]. The deficits observed with ablation of both SPAG16 isoforms, not accounted for by loss of SPAG16L alone, suggest that SPAG16S may play a critical and previously un-described role in spermatogenesis.

We show here that SPAG16S is localized to nuclear subdomains called nuclear speckles. Nuclear speckles are non-nucleolar domains within the nucleus that contain splicing factors as well as transcription factors, RNA processing units, and structural scaffold proteins (reviewed by Lamond and Spector [10]). Though not generally believed to be centers of active transcription, speckles have been implicated as compartments that can provide splicing factor contents to active transcription sites [11], [12]. Speckles are enriched in SC35, which is used as a marker for these distinct domains. SC35 domains have been linked to the development of a cell-type specific genomic organization and to the mapping of distinct “euchromatic neighborhoods” [13]. Though nuclear speckles have been shown to play central roles in management of gene expression, their role in male germ cell differentiation has not been previously reported.

## Results

### Identification of the 5′ UTR of mouse *SPAG16S*

**Figure 1:**
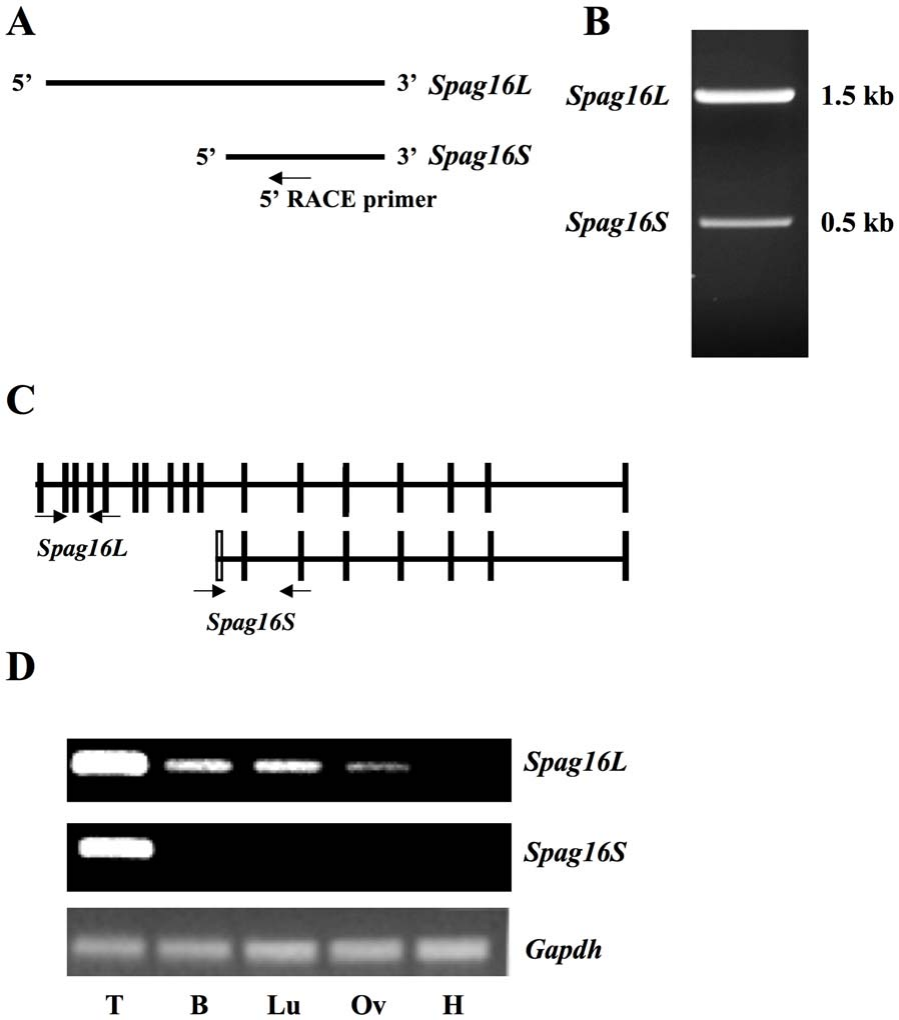
The murine *Spag16* gene encodes two transcripts. *Spag16L* is expressed in all tissues with ciliated cells, while *Spag16S* is expressed only in testis. (A) 5′ RACE was performed with a primer as indicated. (B) Products of 5′ RACE separated on 1% agarose gel. (C) Exon map of *Spag16* transcripts, unfilled box indicating untranslated exon 10a present only in *Spag16S*. Arrows indicate primers used to amplify specifically *Spag16L* or *Spag16S* message. (D) Specific primer sets were used as indicated for PCR amplification of cDNA from adult mouse tissues: Testis (T), Brain (B), Lungs (Lu), Oviduct (Ov), Heart (H).

To identify the 5′ UTR of *Spag16S* mRNA, 5′ RACE was performed with a primer located close to the 3′ end of mouse *Spag16L* mRNA (Fig. 1A). Two PCR products were amplified (Fig. 1B), and each one was cloned into the pCR2.1 Topo TA vector (Invitrogen). 10 clones of each PCR product were sequenced after vector insertion, demonstrating that *Spag16S* sequence is identical to that of *Spag16L* exons 11–17, with the addition of a 5′ untranslated exon, not found in *Spag16L*, named exon 10a (Fig. 1C). This exon 10a is located in the middle of intron 10 of the *Spag16* gene, approximately 50 kb from *Spag16L* exon 10 and 50 kb from *Spag16L* exon 11. Sequencing results demonstrated multiple potential transcription start sites for *Spag16S* transcription; the exon is situated in a TC-rich locus that lacks a standard TATA box (Figure S1).

### *Spag16S* message is expressed only in the testis and male germ cells

SPAG16L is present not only in testis, but also in other murine tissues containing cells with a “9+2” axoneme structure [9], [14]. Primer sets were designed to specifically amplify *Spag16L* (exons 2–4) or *Spag16S* (exons 10a–12) (Fig. 1C). In adult mice, *Spag16L* mRNA was detected in testis, brain, lung, and oviduct; however, *Spag16L* mRNA was not detected in heart tissue (Fig. 1D), which does not contain cells with motile cilia. *Spag16S* mRNA was detected only in testis (Fig. 1D), and in further testing was not detected in kidney, liver, or spleen (data not shown). *Spag16S* expression also appears to be exclusive to males, rather than being a general germ cell factor, as *Spag16S* mRNA was never detected by PCR using up to 10 oocyte equivalents (data not shown).

### Expression pattern of SPAG16S during the first wave of spermatogenesis

**Figure 2:**
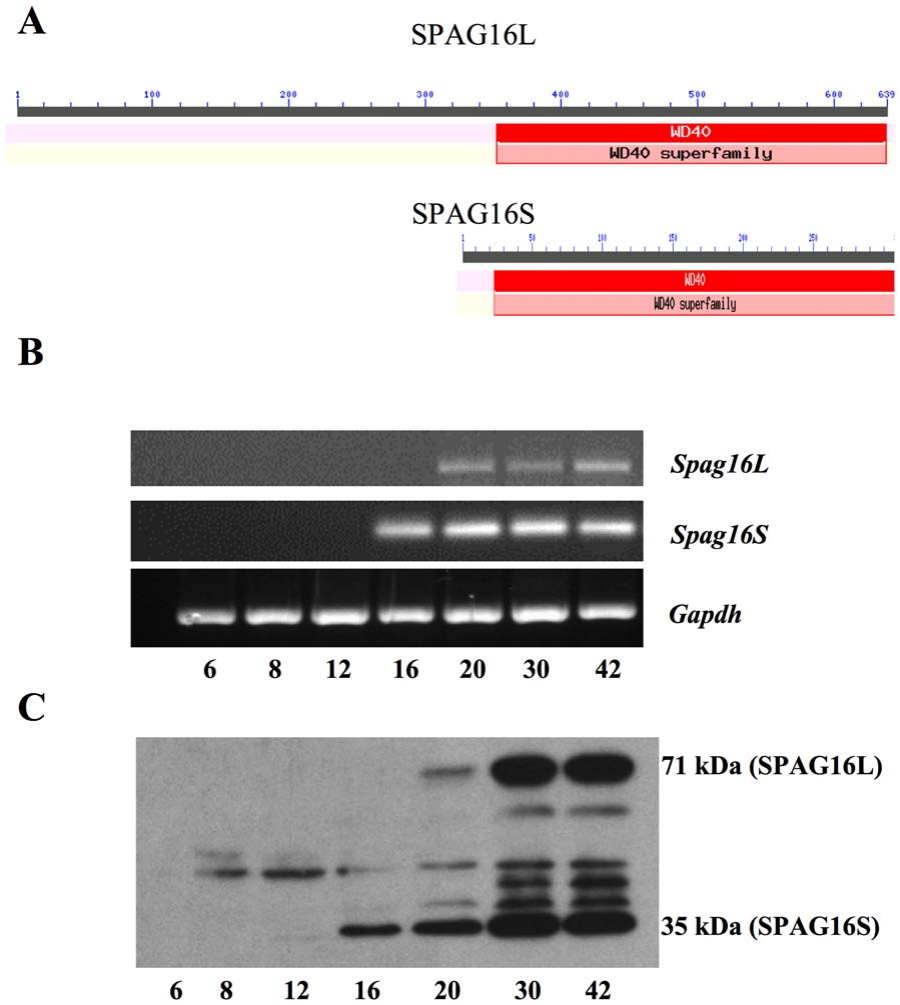
SPAG16 isoforms have identical conserved domains. SPAG16S appears before SPAG16L during the first wave of mouse spermatogenesis. (A) Alignment and conserved domain analysis of SPAG16L and SPAG16S proteins. RNA (B) and protein (C) were isolated from mouse testis at the indicated day (bottom row) after birth. (B) Specific primer sets were used to probe gene expression by PCR of cDNA. (C) Protein samples were separated by SDS-PAGE and probed by Western blotting with a C-terminal SPAG16 antibody that recognizes both SPAG16L (71 kDa band) and SPAG16S (35 kDa band). Additional bands are not specific.

RNA and protein were isolated from mouse testis at 6, 8, 12, 16, 20, 30, and 42 days after birth. cDNA was generated by RT-PCR, and testis extracts were probed by PCR for *Spag16* isoform expression using specific primers. *Spag16S* mRNA was detected at day 16, whereas *Spag16L* mRNA was detected later, at day 20 (Fig. 2B). Western blotting was performed as well, using a polyclonal antibody that recognizes both isoforms of SPAG16 [8]. Consistent with PCR results, SPAG16S protein was detected at day 16, while SPAG16L was detected at day 20 (Fig. 2C). Additionally, both isoforms appeared to be up-regulated at days 30 and 42, consistent with the end of the first wave of spermatogenesis. The immunoreactive bands other than 71 kDa SPAG16L and 35 kDA SPAG16S seen on days 20, 30 and 42 may represent post-translational processing of SPAG16, including proteolytic cleavage of SPAG16L and phosphorylation or other modifications [15].

### Sub-cellular localization of SPAG16 isoforms

**Figure 3:**
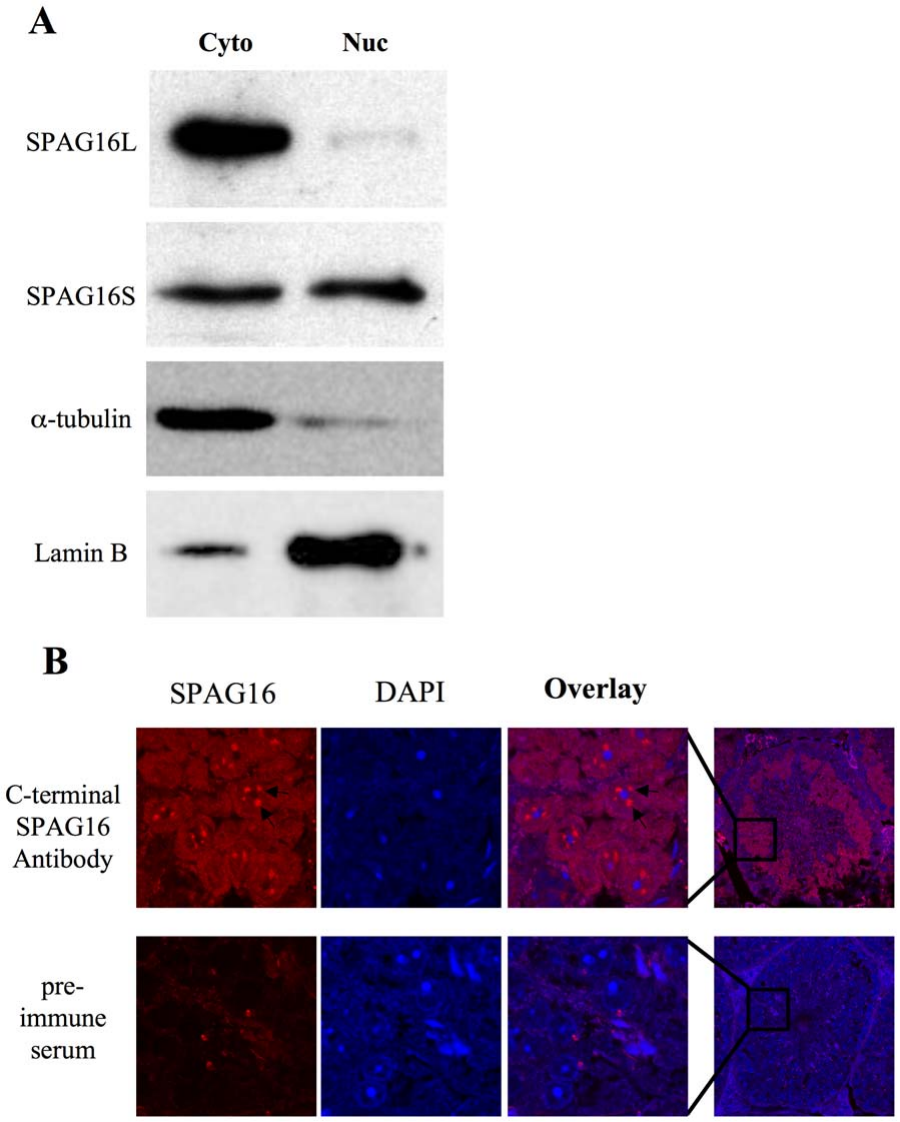
SPAG16L is in the cytoplasm of male germ cells, SPAG16S is in both the nucleus and the cytoplasm. (A) Cytoplasmic and nuclear fractions of adult mouse testis probed by Western blot for SPAG16 (C-terminal antibody recognizing both isoforms) or markers of cytoplasm (α-tubulin) or nucleus (Lamin B). (B) Sections of adult mouse testis immunolabeled with SPAG16 C-terminal antibody or pre-immune serum (negative control). Arrows indicate sample nuclear regions of heightened SPAG16 antibody immunoreactivity.

Cytoplasmic and nuclear fractions of adult mouse testis were isolated, and equivalent amounts of protein from each were probed by Western blot using the C-terminal SPAG16 antibody that recognizes both isoforms. SPAG16L was detected abundantly in the cytoplasm, while SPAG16S was detected in both cytoplasm and nucleus (Fig. 3A). The cytoplasmic localization of SPAG16L is consistent with its identified role as a structural component of the “9+2” axoneme [6], [16], [17]. In order to further characterize the sub-cellular localization of SPAG16S, immunohistochemistry was performed on tissue slices from adult mouse testis. Using the C-terminal SPAG16 antibody, the strongest signal was detected from discrete structures within the nucleus (Fig. 3B, see arrows). Protein expression was most clearly visualized approximately halfway through spermatogenesis, at the round spermatid stage.

**Figure 4 pone-0020625-g004:**
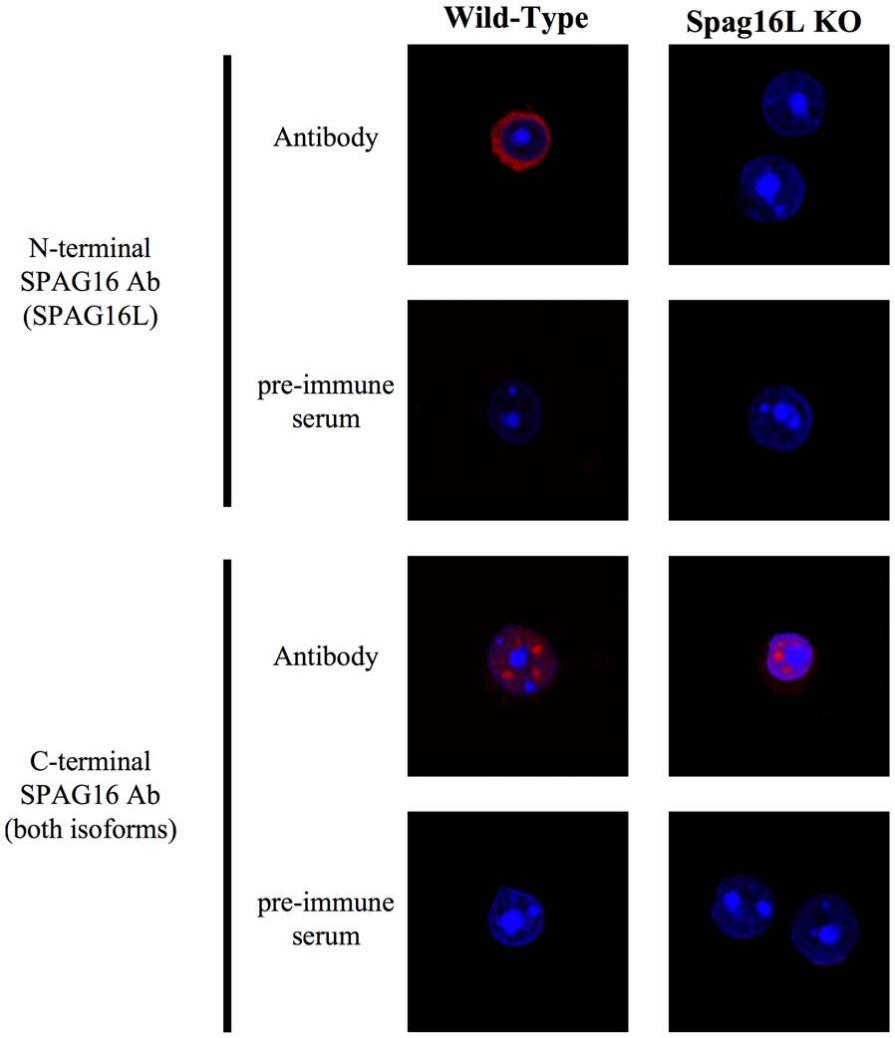
SPAG16S shows enriched nuclear sub-localization. Mixed male germ cells from wild-type or transgenic SPAG16L-KO mice immunolabeled with SPAG16 antibodies (red; N-terminal = SPAG16L only, C-terminal = both isoforms) and nuclear-stained with DAPI (blue). Pre-immune serum for each antibody is included as a negative control.

A mixed population of mouse male germ cells was prepared from adult mouse testis, and immunocytochemistry performed to allow for single-cell imaging to compare with immunohistochemistry results. Consistent with previous data, the C-terminal SPAG16 antibody produced the strongest fluorescence in discrete sub-nuclear, non-nucleolar structures, approximately 2–6 per cell (Fig. 4). The N-terminal SPAG16 antibody, recognizing SPAG16L only, produced an exclusively cytoplasmic signal. Germ cells from SPAG16L-KO mice [9] were isolated and immunolabeled as well, demonstrating the specificity of the antibodies (Fig. 4). As in wild-type germ cells, the C-terminal SPAG16 antibody produced sub-nuclear immunolabelling, which we interpret to represent SPAG16S, while the N-terminal antibody produced no signal, as expected following the deletion of SPAG16L.

### SPAG16S co-localizes with nuclear speckles

**Figure 5:**
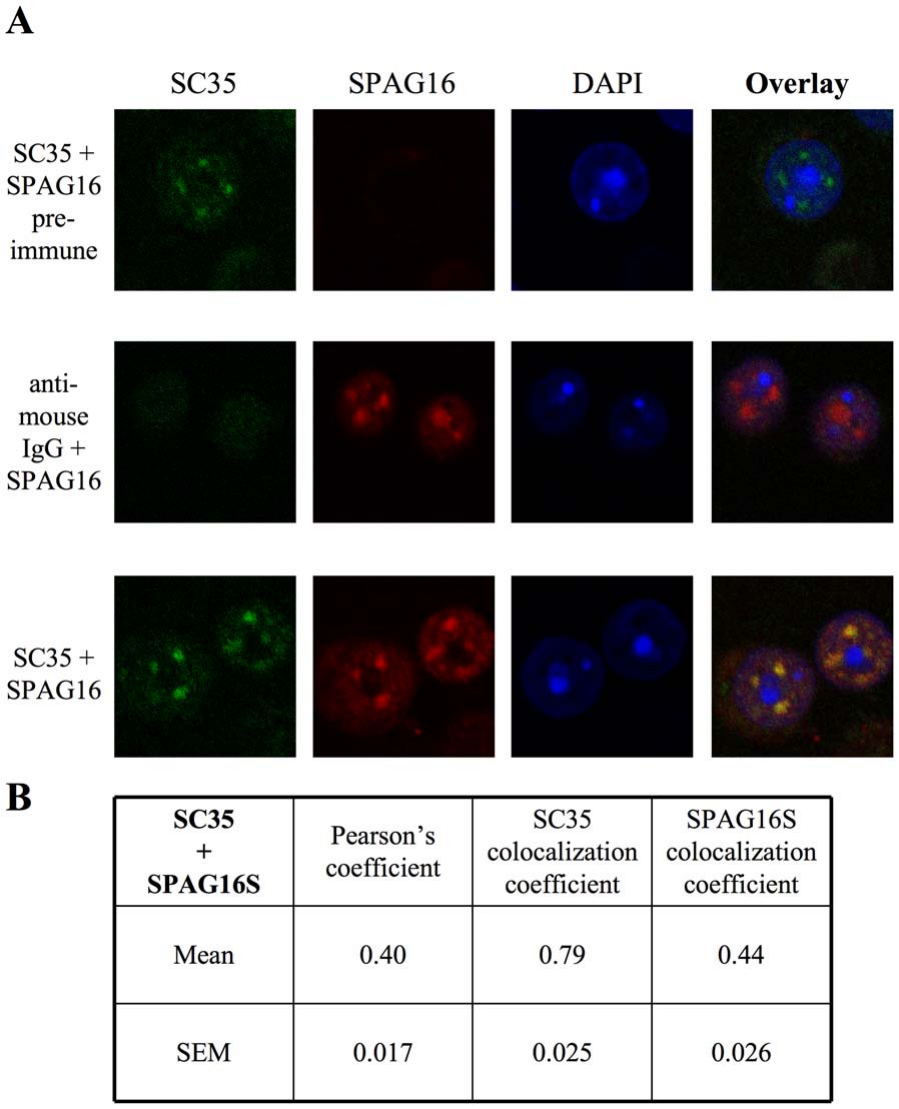
SPAG16S co-localizes with SC35 in nuclear speckles of mouse male germ cells. (A) Mixed male germ cells immunolabeled for SC35 (green) or SPAG16 (red), with DAPI as a nuclear marker. (B) Co-localization analysis of SC35 + SPAG16, *n* = 43.

In order to characterize the discrete nuclear structures identified by immunohistochemical analysis of SPAG16S localization, wild-type germ cells were co-immunolabeled with both the C-terminal SPAG16 antibody and a monoclonal antibody directed against SC35, a marker for nuclear speckles. SC35 and SPAG16S signals strongly overlapped (Fig. 5A). They were determined to have a significant co-localization (Fig. 5B – Pearson's coefficient = 0.40). Analysis demonstrated that 79% of SC35 signal co-localized with SPAG16S, while 44% of SPAG16S signal co-localized with SC35; in other words, most SC35-containing domains also contained SPAG16S, but SPAG16S had a wider distribution as well.

### Role of SPAG16S in SPAG16L expression

**Figure 6:**
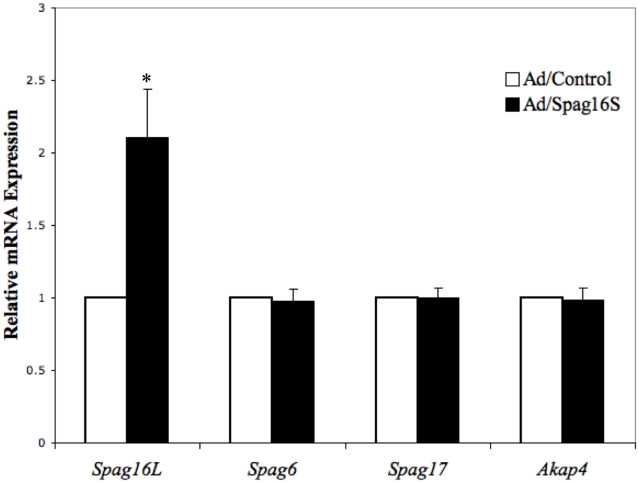
Transduction of SPAG16S induces *SPAG16L* expression in cultured mouse male germ cells. Isolated adult mouse male germ cells were infected with an adenovirus causing SPAG16S transduction or a control adenovirus. Following 48 hours in culture, RNA was isolated and relative expression levels of indicated transcripts were measured. (* p<0.05 compared with Ad/Control).

Given the timing of SPAG16S expression (Fig. 2B–C) and its specific nuclear localization (Fig. 3–5), it was hypothesized that SPAG16S might play a role in regulation of gene expression. Mixed mouse male germ cells were isolated from testis and cultured, with exposure to a SPAG16S-transducing adenovirus or a control adenovirus. Following 48 hour culture, RNA was isolated for analysis of gene expression, and several highly conserved axoneme genes were assessed for mRNA levels. While *Spag6*, *Spag17*, and *Akap4* were unaffected, *Spag16L* message level was significantly increased (Fig. 6).

**Figure 7:**
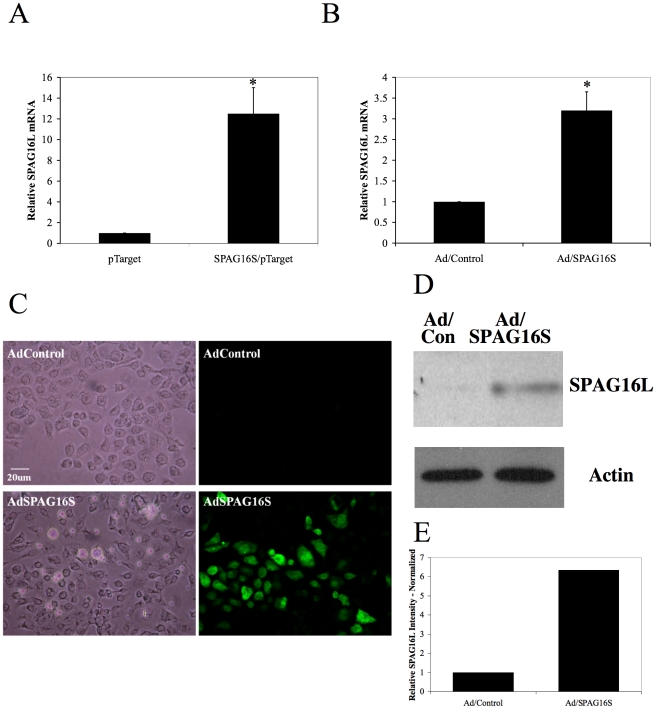
SPAG16S stimulates *Spag16L* mRNA and SPAG16L protein expression in BEAS-2B cells. Analysis of *Spag16L* mRNA expression by real-time PCR in BEAS-2B cells stably expressing SPAG16S (A) or transduced with Ad/SPAG16S (B). (C) Adenovirus-transduced BEAS-2B cells immunolabeled with a C-terminal SPAG16 antibody. (D) Analysis of SPAG16L protein expression by Western blotting in BEAS-2B cells infected by Ad/SPAG16S. This panel demonstrates two independent experiments. (E) Relative intensity of SPAG16L signal in panel D, normalized to Actin loading control for each sample (relative Actin signal for Ad/Control = 47.5%, for Ad/SPAG16S = 52.5%) (* p<0.05 compared with pTarget or Ad/Control).

SPAG16S was also shown to induce SPAG16L expression in BEAS-2B human bronchial epithelial cells. Thiscell line does not express SPAG16L at levels detectable by qPCR or Western blotting (data not shown). Expression of SPAG16S was induced in cultured BEAS-2B cells by plasmid (Fig. 7A) or adenovirus (Fig. 7B), and primers specific for human SPAG16L were used to measure transcript levels, which were normalized to 18S rRNA. Immunocytochemistry performed with the C-terminal SPAG16 antibody confirms that SPAG16 proteins are expressed in the transduced cells, but not in cells exposed to a control adenovirus (Fig. 7C). Following adenoviral transduction, protein was also isolated from cultured BEAS-2B cells, and SPAG16L was demonstrated by Western blot to be present in cells transduced with SPAG16S adenovirus, but not cells transduced with a control adenovirus (Fig. 7D).

**Figure 8:**
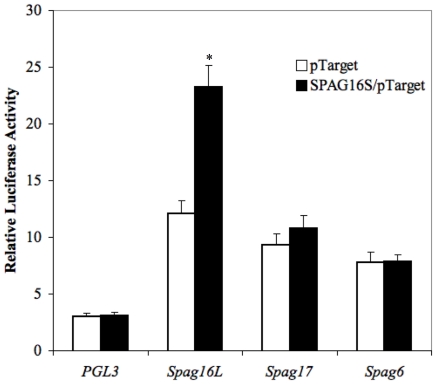
SPAG16S stimulates *Spag16L* promoter activity. Relative luciferase activity, normalized to PGL3 control promoter plasmid co-transfected with pTarget control vector plasmid. Beas-2B cells were co-transfected with indicated promoter/PGL3 constructs (*Spag16L*, *Spag17*, and *Spag6*) and either pTarget or a SPAG16S/pTarget plasmid. Luciferase activity was measured after 48 hours to assess promoter function. (* p<0.05 compared with pTarget control vector co-transfection).

Following the observation that SPAG16L protein and mRNA levels were increased in the presence of SPAG16S, *Spag16L* promoter activity was tested as well. While other axoneme gene promoters (*Spag17*, *Spag6*) did not show significantly altered activity, *SPAG16L* promoter activity was significantly higher in the presence of plasmid-induced SPAG16S when compared to co-transfection with a control empty vector plasmid (Fig. 8).

**Figure 9:**
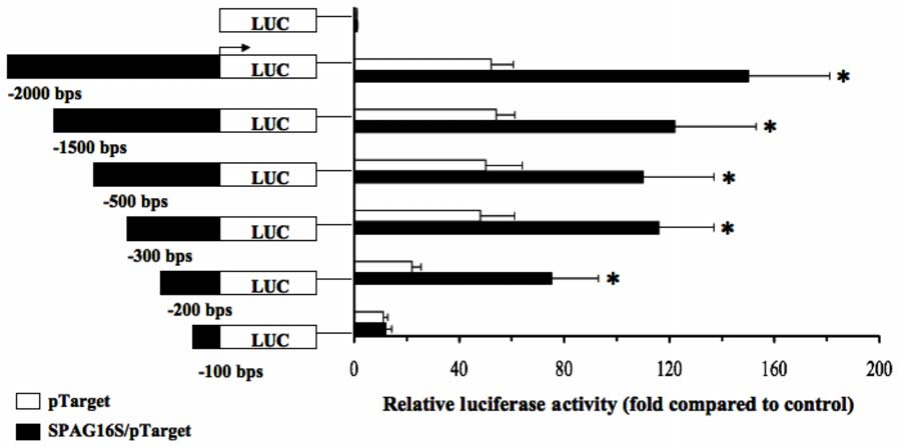
Identification of a *Spag16L* promoter region activated in the presence of SPAG16S. Relative luciferase activity, normalized to PGL3 control promoter plasmid co-transfected with pTarget control vector plasmid. Beas-2B cells were co-transfected with human *Spag16L* promoter plasmids (corresponding to the indicated regions upstream of the transcription start site) and either pTarget control or SPAG16S/pTarget. All promoter constructs except the −100 bp promoter demonstrated significantly increased activity in the presence of SPAG16S co-transfection. (* p<0.05 compared with pTarget co-transfection for a given promoter).

In order to identify a specific region within the *SPAG16L* promoter showing increased activity with SPAG16S expression, luciferase plasmid constructs were made with progressively shorter sequences of the *SPAG16L* proximal promoter, ranging from 2 kb to 100 bp upstream of the transcription start site. Promoters ranging from 2 kb to 200 bp upstream of the transcription start site demonstrated significantly higher activity when co-transfected with a SPAG16S plasmid, but a 100 bp upstream *SPAG16L* promoter did not show increased activity in the presence of SPAG16S plasmid (Fig. 9).

## Discussion

Flagella and cilia are strongly conserved structures, having maintained a highly specific structure and function throughout eukaryotic life, from unicellular protists to mammals [18], [19]. It has been suggested that molecular structures of each component in the axoneme are not always conserved, but protein *domains* within axoneme structures are preferably conserved, allowing for overall morphological and functional conservation [20], [21]. Although the *Chlamydomonas PF20* gene encodes only one transcript, its mouse orthologue encodes two mRNAs for two proteins, SPAG16L and SPAG16S, each of which have conserved WD-repeat domains. To study the expression pattern of the two mRNAs and proteins, testicular total RNAs and proteins were isolated from mice at different ages. Given that *Spag16S* harbors an untranslated exon 10a – which is not present in Spag16L – a *Spag16S*-specific forward primer was designed to selectively amplify the *Spag16S* transcript. Both SPAG16S mRNA and protein are expressed earlier than *SPAG16L* message and SPAG16L protein. For both SPAG16L and SPAG16S, mRNAs and proteins appear simultaneously (SPAG16S at day 16, followed by SPAG16L at day 20), suggesting that there is no translation delay [22], [23]. Furthermore, *Spag16L* is expressed in other tissues, such as brain and lung, all of which contain motile cilia [16], but *Spag16S* is expressed only in the mouse testis, indicating that *Spag16S* is a testis-specific gene product. Moreover, *Spag16S* sequences identified in GenBank in a variety of mammalian species (Mouse: NM_025728.3; Rat: BC158602.1; Human: EF591776.1) have each been detected from testis samples.

Testis-specific genes can be grouped into three clusters. Homologous genes are those which are expressed only in spermatogenic cells, but which are closely related to genes expressed in somatic cells, and are often members of gene families. Unique genes are those without significant similarity to any other genes in the genome. Variant transcripts are transcribed from genes also expressed in somatic cells but are often smaller or larger than their somatic cell counterparts. They are the result of the utilization of one or more alternate transcriptional start sites, splice sites, or polyadenylation signals. Since spermatogenesis is a highly coordinated process, different genes are expressed during the three different stages of spermatogenesis, and gene expression can be used as a tool to assess progression through the stages of spermatogenesis [24]–[26]. There are genes that have been shown to be expressed solely in the mitotic phase (spermatogonia) [27]. Some are expressed during the meiotic phase (spermatocyte) [28] and others during the post-meiotic phase in spermatids [29]. Some of these genes are expressed solely in germ cells, while others are also expressed in somatic cells as well.

It appears that the *Spag16S* transcript is unique, as no related transcripts were identified in somatic cells. It is unlikely to be a spliced isoform of the *Spag16L* transcript, since it is detected in testis of mice homozygous for a mutation that ablates the Spag16L mRNA [9]. Similarly, evidence suggests that the SPAG16S protein is not a processed form of SPAG16L, since the SPAG16S protein is present in SPAG16L-deficient mice, and the expression of SPAG16S occurs earlier than SPAG16L (Figure 2).

The mouse *Spag16S* 5′-UTR and upstream putative promoter region are highly conserved in rat (see Figure S1). It is worth noting that only a single *Spag16* transcript has been identified in rat (BC158602.1), and its coding sequence corresponds to the region shared by both mouse *Spag16* isoforms. However, the rat *Spag16* mRNA includes a 410 bp 5′-UTR that is nearly identical to the mouse exon 10a plus its immediate upstream genomic sequence [30]. Moreover the rat *Spag16* transcript was identified specifically in testis, leaving open the possibility that a *Spag16L*-like transcript may be likely to exist in testis and other tissues.

Different subcellular localization of SPAG16L and SPAG16S suggests that the two proteins play different roles. This was subsequently confirmed by two knockout models generated previously in our laboratory. SPAG16L regulates ciliary motility [9], while SPAG16S controls spermatogenesis [8]. Loss of both SPAG16 isoforms produced a non-transmitted allele and led to significant arrest and cell death at the round spermatid stage in chimeric male mice carrying the mutant gene. While homozygosity for a SPAG16L-deletion mutation produced male infertility, the mutant allele was transmitted in the chimeric and heterozygous states, and the perturbation of spermatogenesis observed with total SPAG16 knockout was not seen. It has been observed that genetic mutations contributing to male infertility are often discovered to take effect through unexpected or unexplored cellular pathways. Thus, while *Spag16* is known to be important to the axoneme through SPAG16L, the existence of an additional protein of unknown – and potentially more essential – function is consistent with published findings [31].

Both SPAG16L and SPAG16S contain 7 WD-repeat domains, semi-conserved 40 amino acid-regions ending with tryptophan-aspartate (W-D). These regions are known to mediate protein-protein interactions by giving rise to a ß–propeller tertiary structure. By interacting with effector partners, WD repeat proteins have been shown to play important roles in an extensive variety of cellular activities, including cell division, gene transcription, mRNA modification, and transmembrane signalling (reviewed in [32], [33]). More recently, it has been reported that WD domains may also mediate specific processing of small RNAs [34] and direct binding of RNAs [35], further diversifying the range of potential roles these structures can play in eukaryotic cells. Because SPAG16S contains no obvious nuclear localization signals, its importation into the nucleus may be effected by it association with other proteins including transcription factors or nuclear speckle components.

We have demonstrated that while SPAG16L is exclusively in the cytoplasm of male germ cells – consistent with its structural role in the axoneme – SPAG16S is present in both nucleus and cytoplasm, and exhibits enhanced sub-localization within the nucleus. A monoclonal antibody directed against SC35, a canonical marker for nuclear speckles [36], [37], maps to the same nuclear regions that show enhanced SPAG16S signal, suggesting that SPAG16S is located in nuclear speckles of male germ cells, specifically at the round spermatid stage. Nuclear speckles are enriched in splicing-related factors, and though they are not active centers of transcription [10], they have been shown to associate with active alleles [38]–[41].

The role of nuclear speckles has yet to be described in mammalian spermatogenesis. Though somatic cells nuclei contain 30–50 nuclear speckles, these structures are known to condense following inhibition of transcription [42]; thus, the presence of only a few, larger speckles at the round spermatid stage is consistent with the down-regulation of transcription during sperm development. The unique nature of transcript and protein packaging during nuclear condensation in spermatogenesis suggests the intriguing possibility that nuclear speckles may play a distinct role in this process, and that SPAG16S may be a germ cell-specific factor necessary to guide this process.

*SPAG16L* mRNA and SPAG16L protein are up-regulated *in vitro* in the presence of SPAG16S, suggesting that one role of SPAG16S involves regulation of the SPAG16L isoform. These data are consistent with the observation that SPAG16L appears after SPAG16S. We have also shown that the *Spag16L* promoter displays significantly enhanced activity in the presence of SPAG16S.

While we cannot rule out the possibility that SPAG16S is involved in SPAG16L regulation through transcript processing events in nuclear speckles, these data strongly suggest that a primary responsibility of SPAG16S is activation of *SPAG16L* transcription. Since SPAG16S does not have an identified DNA-binding domain, it is unlikely to directly bind the *Spag16L* promoter, but rather to effect this interaction by binding with one or several protein partners. WD-repeat proteins are known to interact dynamically and reversibly with multi-protein complexes, thus identification of SPAG16S-associating structures presents a challenging but enticing area for future study.

In summary, SPAG16S is a testis-specific protein found in nuclear speckles that appears to regulate spermatogenesis by controlling testis-specific target gene expression, one of the target genes being *Spag16L*. Thus, the murine *Spag16* gene has dual functions. It encodes a structural protein at the axoneme, which is essential for sperm motility, and a nuclear speckle-associated factor that regulates *Spag16L* gene expression. To the best of our knowledge, this is the first example of a gene's evolution conferring the ability to regulate its own, conserved products.

## Materials and Methods

### Ethics Statement

No human or primate subjects were used in this work. All rodent work was approved by Virginia Commonwealth University's Institutional Animal Care & Use Committee (protocol permit #AM10297) in concordance with all federal and local regulations regarding the use of non-primate vertebrates in scientific research. Research animals were humanely housed and care was taken to prevent undue distress.

### 5′ Rapid amplification of cDNA ends (5′ RACE)

5′ RACE was carried out to define the 5′ non-translated region sequence and transcriptional start site of the mouse Spag16S mRNA using mouse Marathon cDNA amplication kit (Clontech) according to the manufacturer' instructions. Briefly, a reverse primer was designed within the coding region of Spag16 (5′-AGAAGCCACGAAGTCACCACAGGAGT-3′) and used together with the Marathon cDNA adaptor primer to generate 5′-RACE products. The smaller product was cloned into the pCR2.1-TOPO TA vector and subjected to DNA sequence analysis.

### Conserved domain analysis

Mouse SPAG16S (NP_080004.1) and SPAG16L (NP_083436.2) protein sequences were analyzed using NCBI Conserved Domains tool [43], [44].

### Antibodies

SPAG16-specific antibodies directed against the common C-terminus (SPAG16L amino acids 330–639) and the SPAG16L-specific N-terminus (amino acids 1–212) have been previously described [7], [8]. α-tubulin (cytoplasmic marker) and Lamin B (nuclear marker) were from Santa Cruz Biotechnology. SC35 was purchased from Sigma-Aldrich.

### Microscopy and colocalization analysis

Confocal laser scanning microscopy was performed using a Leica TCS-SP2 AOBS (Leica Microsystems). Colocalization calculations were performed using Volocity Quantitation software (Perkin-Elmer).

### Mixed germ cell preparation

A male adult mouse was anesthetized and euthanized, and the testis decapsulated and placed in 5 mL PBS. Collagenase IV (Sigma-Aldrich) was added to a final concentration of 0.5 mg/mL and DNase I (Sigma-Aldrich) to a final concentration of 1.0 mg/mL. Testis solution was incubated 30 min at 32°C to dissociate cells, then centrifuged 5 min at 1000 rpm. Cells were fixed by 15 min incubation in 1% paraformaldehyde/PBS at room temperature, then washed three times with PBS. Prior to plating, cells were re-suspended in 12.5 mL PBS and 50 µL of cell suspension was spread on SuperFrost/Plus microscope slides (Fisher Scientific) and allowed to air-dry. Slides were used immediately for immunocytochemistry or stored at −80°C until use.

### Cell fractionation into nuclei and cytoplasm

Freshly isolated mouse male germ cells (see germ cell slide preparation protocol) were separated into nuclear and cytoplasmic fractions using a Nuclear/Cytosol Fractionation Kit (BioLine, Inc.) per manufacturer's instructions.

### Western blotting analysis

Equal amounts of protein (50 µg/lane) were heated to 95°C for 10 minutes in 4× sample buffer, loaded onto 10% sodium dodecyl sulfate-polyacrylamide gels, electrophoretically separated, and transferred to PVDF membranes. The membranes were blocked and then incubated with primary antibody at 4°C overnight. After being washed, the blots were incubated appropriate HRP-conjugated secondary antibody (GE Healthcare UK) for 1 hour at room temperature. After washing, protein was detected with Super Signal Chemiluminescent Substrate (Pierce). Densitometry performed using ImageJ [45].

### Immunocytochemistry and immunohistochemistry

Slides were first blocked 1 hour at room temperature with 10% goat serum/PBS. Following overnight incubation with primary antibody (diluted 1∶100–300 in blocking medium) at 4° C, slides were washed with PBS and incubated 1 hour at room temperature with Alex 488-conjugated anti-mouse IgG secondary antibody (1∶500; Jackson ImmunoResearch Laboratories) or Cy3-conjugated anti-rabbit IgG secondary antibody (1∶1000; Jackson ImmunoResearch Laboratories). Following secondary antibody incubation, slides were washed in PBS and sealed using VectaMount with DAPI (Vector Laboratories).

### Generation of adenovirus to express SPAG16S

Adenovirus expressing SPAG16S (AdSpag16s) was generated with AdEasyTM Adenoviral Vector System (Stratagene) following the instruction manual. Briefly, mouse Spag16S cDNA was subcloned into adenovirus shuttle vector pShuttle-CMV and the cDNA was transferred into pAdEasy-1 virus genome by means of homologous recombination in an adenovirus packaging cell line HEK-293 cells (ATCC; Manassas, VA). The expression of SPAG16S was tested with western blotting using proteins from COS-1 cells (ATCC) and BEAS-2B cells (ATCC) infected with AdSpag16S or control Ad virus. The AdSpag16S and control Ad virus were amplified and titered in the Macromolecule Core Facility of Virginia Commonwealth University.

### Generation of plasmid to express SPAG16S

Mouse testis cDNA was used as a template to amplify *Spag16S* sequence by PCR. Amplified PCR product was cloned in a pCR2.1 Topo TA vector for sequencing, then released by restriction enzyme digestion and ligated in a pTarget vector. Primers used: Forward (*BamHI*): 5′-GGATCCCCTGTAGATATGCAACCAGATCC-3′; Reverse (*SalI*): 5′-GTCGAC GCTGATCAGATCCACAACCGAATG-3′.

### RT-PCR and real-time PCR

Total RNA was isolated from cultured cells and indicated tissues with Trizol (Life Technologies, Inc., Grand Island, N.Y.), the RNA was reversed transcribed, and the cDNAs were used for RT-PCR or real-time PCR.

For real-time PCR, cDNAs from BEAS-2B cells transfected with pcDNA3 or Spag16S/pcDNA3 plasmids or infected with AdSpag16S or control Ad virus were utilized for PCR. Primers were designed for detection of human *SPAG16L* using the software from GeneScript Corporation (http://www.genscript.com/). Real-time PCR reactions were carried out using the 2× SYBR green master mix (BioRad). Akap4, Spag17, and Spag6 primers have been previously reported [46].

(human) *SPAG16L* – Forward: 5′-TTCAGACTGCTGCTTCCATC-3′; Reverse: 5′-TCGCCTGTACATAGATCCCA-3′

(mouse) *Spag16L* – Forward: 5′-AGCAAGCCAGAGACATCCAT-3′; Reverse: 5′-CCAGAAATCTTCCCAACAGC-3′

(mouse) *Spag16S* – Forward: 5′-CTCTGACACAATGAGTATGG-3′ (exon 10a); Reverse: 5′-CTACAGGAAATTCTGAATCC-3′ (exon 11)

*18S* – Forward: 5′-GGCCCTGTAATTGGAATGAGTC-3′; Reverse: 5′-CCAAGATCCAACTACGAGCTT-3′

### Luciferase promoter activity assay

Promoter plasmids were designed using a PGL3 basic vector (Promega). For each experiment, BEAS-2B cells were co-transfected with empty vector or promoter plasmid and a control (pTarget - Promega) or test (SPAG16S) plasmid using Fugene 6 transfection reagent (Roche) then cultured 48 hours. Cells were lysed using 1× passive lysis buffer according to manufacturer's instructions (Promega) and luciferase activity measured using freshly-prepared reagents. Data are represented as relative fold difference from PGL3 control promoter co-transfected with control pTarget vector plasmid.

### Generation of promoter constructs for promoter function assays

Promoter sequences were amplified by PCR from mouse DNA and sub-cloned in a PGL3 basic vector after sequences were confirmed by first cloning in a Topo Vector (Invitrogen) for sequence analysis. Primers were designed to specifically amplify regions upstream of the transcription start site (Fig. 9). 2 kb, 1.5 kb, and 0.5 kb promoter constructs were previously generated in our lab [46]. 0.3 kb, 0.2 kb and 0.1 kb promoter constructs were generated as described using a common reverse primer (5′-CTCGAGGCTTGCAACTGCGGCCCCTCGGTGCC-3′) wit the following forward primers:

0.3 kb *Spag16L* –5′-GGTACCCGCAAGCAAGCAAGCAAGCAAGCAAGC-3′
0.2 kb *Spag16L* – 5′-GGTACCGGTTCTGGGCTTCAGGTCTGCAGTCC-3′
0.1 kb *Spag16L* – 5′-GGTACCCGCTTGACCGGGGCCTTTTGGTGC-3′

### Statistical Analysis

Statistical analyses were performed in Microsoft Excel using a t test.

## Supporting Information

Figure S1 **Mouse and Rat** ***Spag16S*** **putative promoter and 5′-UTR regions.** Sequences of mouse and rat transcription start sites and putative upstream promoter regions. Transcription start sites as noted in red correspond with GenBank sequences (mouse – NM_025728.3; rat – BC158602). Upstream genomic regions are also as noted in GenBank (mouse – AY742710.2; rat – NC_00508: 68851813–68853621). Alignment analysis performed using MacVector v10.6.
(TIF)
