# Supplementary figures and images for: Spag16, an Axonemal Central Apparatus Gene, Encodes a Male Germ Cell Nuclear Speckle Protein that Regulates SPAG16 mRNA Expression
Source: PLoS One. 2011 May 31;6(5):e20625. doi: 10.1371/journal.pone.0020625 (PMC3105110; doi:10.1371/journal.pone.0020625)

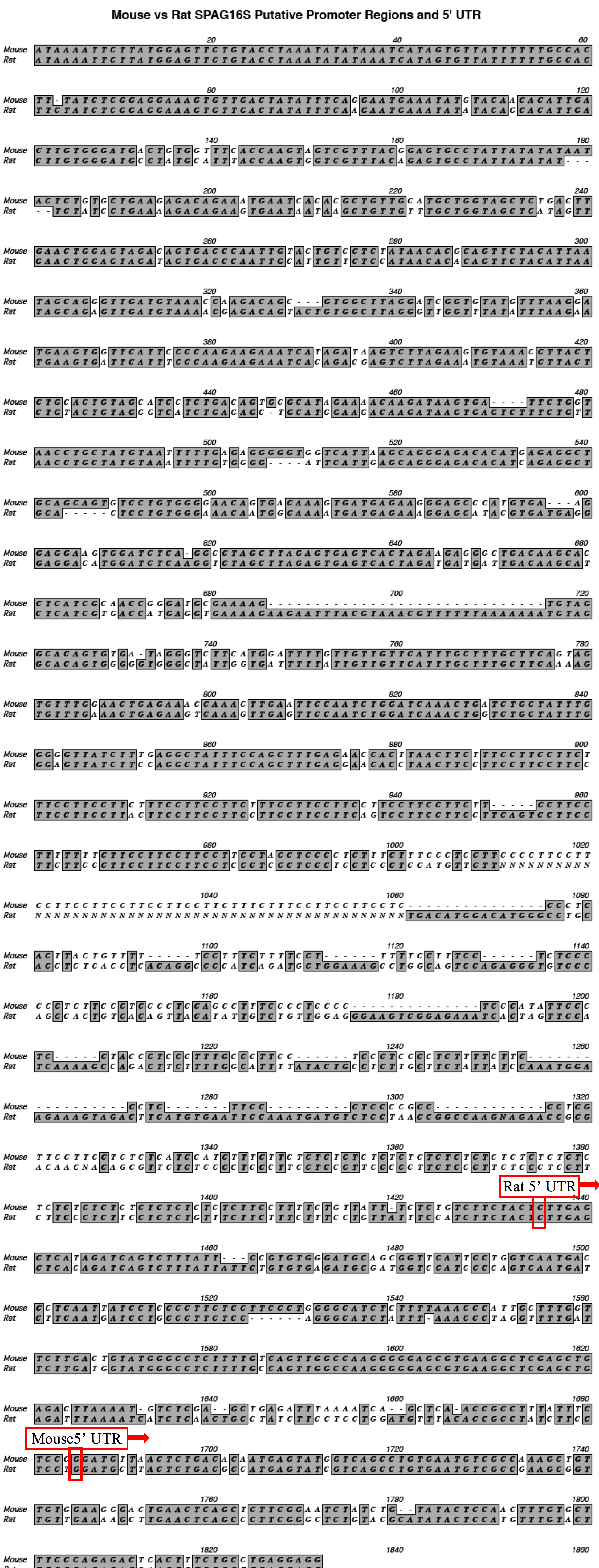

Supplement: Figure S1 — Mouse and Rat Spag16S putative promoter and 5′-UTR regions. Sequences of mouse and rat transcription start sites and putative upstream promoter regions. Transcription start sites as noted in red correspond with GenBank sequences (mouse – NM_025728.3; rat – BC158602). Upstream genomic regions are also as noted in GenBank (mouse – AY742710.2; rat – NC_00508: 68851813–68853621). Alignment analysis performed using MacVector v10.6. (TIF) [file pone.0020625.s001.tif]
